# Some Aspects of Military Ophthalmology

**Published:** 1919

**Authors:** 


**Some Aspects of Military Ophthalmology.** By S. HANFORD McKEE. *British Medical Journal,* Sept. 28, 1918.

The military point of view should teach a medical officer " to size up " his patient, and considerable latitude should be allowed the military ophthalmologist as to who " sees to shoot " and who " sees enough for ordinary purposes ".

Unless a soldier's vision is improved considerably by glasses he will not wear them, so there is only one reason which warrants prescribing glasses for a soldier; namely, that, by prescribing glasses, you change him from an unfit to a fit soldier. It is a grave mistake to prescribe them, for when you do you supply him with an excuse which he always has for lining up on sick parade. It is to be remembered that, in a soldier, complaints of headache or pains of any sort are not reliable symptoms of disease.

During the examination for refraction a variety of defects are found, prominent among them *amblyopia ex anopsia*. The author has not seen an epidemic of conjunctivitis among soldiers, and very few cases of gonorrheal ophthalmia. Trachoma has almost disappeared as a military disease. Among military symptoms of disease night blindness takes a prominent part. It seems that we may well attribute this complaint or disease in a certain number of cases to exposure, hard work, and great fatigue. Syphilis of the eye is quite frequent. Sympathetic ophthalmia is very rare.

The concussion following modern explosives leads to a great variety of fundus lesions. The injuries are caused by the violence communicated through the bones of the skull, the bones of the orbit, or by air vibration — windage. One of the commonest of these lesions is a condition of traumatic retino-choroiditis, characterized by diffuse cloudiness of the retina, numerous small exudates in the choroid, and fine dust-like opacities of the vitreous. It is identical with retino-choroiditis of secondary syphilis.

One reason only should form the basis of all military operative work — necessity. Dacryocystitis is not infrequently met with. The author very strongly recommends the West operation.

Major Waldron has modified Esser's method of epidermic inlay for remedying distortions of the lid. He makes an incision in the conjunctival sac instead of through the eyelid skin. Major Gillies buries the graft covered mould in the subcutaneous tissues of the eyelid through an incision in the skin, and removes it through that incision. The second method may be termed an epithelial overlay.

